# Perillaldehyde-Elicited Inhibition of Ochratoxin A Production by *Aspergillus carbonarius*

**DOI:** 10.3390/toxins17110530

**Published:** 2025-10-29

**Authors:** Dongmei Jiang, Liuqing Wang, Nan Jiang, Jiaqi Yan, Jingzhi Mei, Meng Wang

**Affiliations:** 1 Institute of Quality Standard and Testing Technology, Beijing Academy of Agriculture and Forestry Sciences, Beijing 100097, China; jiangdm@iqstt.cn (D.J.); wanglq@iqstt.cn (L.W.); jiangn@iqstt.cn (N.J.); meijingzhi1024@163.com (J.M.); 2 Laboratory of Quality & Safety Risk Assessment for Agro-Products (Beijing), Ministry of Agriculture and Rural Affairs, Beijing 100097, China; 3 College of Horticulture, China Agricultural University, Beijing 100193, China; yanjiaqi@cau.edu.cn; 4 College of Food Science and Nutritional Engineering, China Agricultural University, Beijing 100183, China

**Keywords:** mycotoxin, bioactive compound, antifungal, *Aspergillus*, molecular mechanism

## Abstract

Ochratoxin A (OTA) is a major mycotoxin contaminant in grapes and their products, and *Aspergillus carbonarius* is its main producer. Controlling the growth of *A. carbonarius* is therefore critical for mitigating OTA contamination. Plant-derived perillaldehyde, with good antifungal activity and safety, has garnered growing attention. However, current understanding of how perillaldehyde affects *A. carbonarius* growth and OTA production remains poorly characterized. In this study, we systematically investigated the antifungal and antimycotoxigenic effects of perillaldehyde against *A. carbonarius* and explored the underlying mechanisms. The results showed that perillaldehyde could alter the mycelial morphology and damage the cell integrity of *A. carbonarius*. Additionally, perillaldehyde could diminish the total antioxidant capacity and impair the energy metabolism of *A. carbonarius*. Transcriptome analysis showed that the expressions of all the known conserved OTA biosynthetic genes and two OTA transport-related genes were significantly down-regulated, indicating that perillaldehyde could directly inhibit their expression. In conclusion, perillaldehyde can significantly inhibit OTA production by directly disrupting OTA biosynthesis and transport and inhibiting the growth of *A. carbonarius*. Thus, perillaldehyde has the potential to be used as a natural fungicide or alternative food preservative in grapes and their products, owing to its strong antifungal and antimycotoxigenic effects on *A. carbonarius*.

## 1. Introduction

Exposure to ochratoxin A (OTA)–a polyketide-derived mycotoxin–can induce diverse toxicological effects, including nephrotoxicity, hepatotoxicity, and immunotoxicity, as well as teratogenic, mutagenic, and carcinogenic consequences [1,2,3,4]. The International Agency for Research on Cancer (IARC) classified OTA as a potential human carcinogen in 1993 [1]. OTA is a major contaminant of various agricultural products, such as grains, grapes, as well as their products [5]. OTA represents the most significant mycotoxin contaminant in grapes and grape-derived products, with *Aspergillus carbonarius* being the predominant OTA-producing species [6]. Given the critical role of fungal growth suppression in mitigating mycotoxin contamination in agricultural commodities, implementing effective strategies to control *A. carbonarius* proliferation is essential for reducing OTA pollution.

Conventional mycotoxin control has primarily relied on chemical fungicides. However, growing concerns over their associated risks, such as environmental contamination, toxic residue accumulation, and drug resistance, have spurred increasing interest in plant-derived antimicrobials. These natural alternatives, particularly plant essential oils and their bioactive constituents, offer distinct advantages such as broad-spectrum antimicrobial activity, environmental biodegradability, high volatility, and minimal residue persistence. These inherent characteristics position them as promising eco-friendly substitutes for synthetic fungicides in mycotoxin management strategies [7].

Perillaldehyde, a monoterpenoid compound derived from Perilla oil, is an eco-friendly and safe natural antimicrobial agent approved for use as a food additive [8,9]. This bioactive component demonstrates potent broad-spectrum antifungal activity against multiple fungal species, including *Aspergillus niger*, *Aspergillus flavus*, *Alternaria alternata*, and *Candida albicans* [10,11]. Notably, studies have confirmed its dual inhibitory effect on both *A. flavus* growth and aflatoxin biosynthesis [12,13]. The antifungal mechanism involves disruption of cellular membrane integrity through inhibition of membrane constituent synthesis and direct membrane disintegration [14,15]. Despite these documented effects, the efficacy of perillaldehyde against *A. carbonarius* growth and OTA production remains poorly characterized, with limited data available from either *in vitro* or *in vivo* studies. This limitation not only restricts the application of perillaldehyde in fruit and vegetable preservation but also creates a critical gap in understanding how perillaldehyde modulates the intrinsic antifungal pathways of mycotoxin-producing fungi and its potential to interfere with OTA biosynthesis.

Extensive research has elucidated several key enzymes involved in OTA biosynthesis, including polyketide synthase (OTApks, OtaA), non-ribosomal peptide synthase (OTAnrps, OtaB), cytochrome P450 monooxygenase (P450, OtaC), halogenase (OTAhal, OtaD), and bZIP transcription factor (bZIP, OtaR1) [16]. Notably, a recent discovery revealed the critical role of a SnoaL cyclase (OTAcyc, OtaY) domain located between the OtaA and OtaB in facilitating polyketone cyclization during the initial stage of OTA biosynthesis [17]. Despite these advances, a significant knowledge gap remains regarding natural inhibitors of this pathway. Importantly, no studies to date have investigated the inhibitory effects or molecular mechanisms of perillaldehyde against *A. carbonarius* growth and OTA biosynthesis. This study presents the first comprehensive investigation that integrates physiological, biochemical, and metabolic analyses with transcriptomic profiling to systematically elucidate the mechanisms by which perillaldehyde suppresses *A. carbonarius* proliferation and disrupts OTA biosynthesis. Our novel findings will enrich the current understanding of how plant-derived compounds regulate the metabolism of mycotoxin-producing fungi, and establish a critical theoretical framework for applying perillaldehyde as an eco-friendly antifungal agent to mitigate OTA contamination in grapes and grape-derived products.

## 2. Results and Discussion

### 2.1. Inhibitory Effects of Perillaldehyde on Mycelial Growth and OTA Production

**Figure 1:**
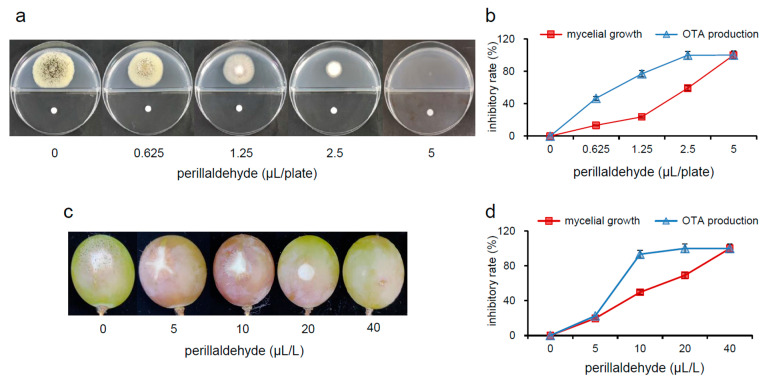
Inhibitory effects of perillaldehyde on mycelial growth of *Aspergillus carbonarius* and OTA production *in vitro* and *in vivo*. (**a**) Morphology of *A. carbonarius* on PDA medium. (**b**) Inhibition rates of fungal growth and OTA production at different concentrations of perillaldehyde (0, 0.625, 1.25, 2.5, and 5 μL/plate) on PDA medium. (**c**) Morphology of *A. carbonarius* in grapes. (**d**) Inhibition rates of fungal growth and OTA production at different concentrations of perillaldehyde (0, 5, 10, 20, and 40 μL/L) in grapes. OTA: ochratoxin A. The inhibitory rate data are expressed as the mean ± standard error (*n* = 3).

Our results demonstrated dose-dependent inhibition of *A. carbonarius* mycelial growth by perillaldehyde on potato dextrose agar (PDA) medium (Figure 1a). We found that 0.625, 1.25, 2.5, and 5 µL/plate of perillaldehyde significantly reduce the mycelial growth of the fungi, with the reduction percentage varying from 13.3% to 100% (Figure 1b). Since 5 µL/plate perillaldehyde completely suppresses the mycelial growth of *A. carbonarius*, this concentration was defined as the minimum inhibitory concentration (MIC) of perillaldehyde against its mycelial growth. Previous research has reported that citral and eugenol are able to inhibit the proliferation of *A. carbonarius*, and the inhibitory effects depend on the concentration used [18,19]. Perillaldehyde also exhibited dose-dependent inhibition performance in the growth of *A. flavus*, *A. niger*, *A. oryzae*, and *Alternaria alternate* [12]. In addition, perillaldehyde exhibited a much stronger inhibition of OTA production over mycelial for all concentrations in the experiment, with the inhibition rates varying from 46.7% to 100%. For example, 1.25 μL/plate of perillaldehyde results in a 77.0% reduction in OTA production, but only a 23.7% decrease in mycelial growth. OTA suffered nearly complete inhibition at 0.5×MIC, but mycelial growth was inhibited by only 59.2%. Comparable *in vivo* results were acquired from grapes infected by *A. carbonarius*. Perillaldehyde exhibits the capacity to restrain the mycelial growth of *A. carbonarius* (Figure 1c) and OTA production (Figure 1d) in grapes in a dose-dependent manner (0, 5, 10, 20, and 40 μL/L concentrations). The inhibition rates of mycelial growth range from 19.7% to 100%. The mycelial growth in grapes was completely suppressed when 40 µL/L of perillaldehyde was employed as the MIC, while OTA production was completely inhibited at 0.5×MIC. Therefore, irrespective of whether perillaldehyde is applied to PDA medium or to grapes, OTA production is significantly inhibited compared to mycelial growth. Thus, OTA production does not decrease just because of the inhibition of mycelial development.

### 2.2. Effects of Perillaldehyde on Mycelial Morphology of A. carbonarius

**Figure 2:**
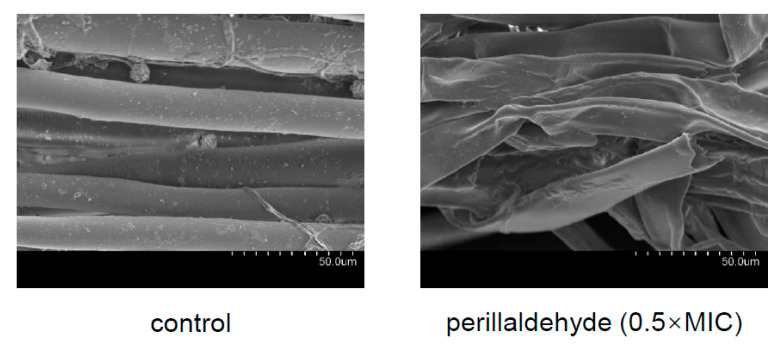
Morphology of *A. carbonarius* under scanning electron microscopy (SEM). MIC: minimal inhibitory concentration.

Control group mycelia exhibited normal morphology, displaying smooth, uniform, and continuous hyphal structures (Figure 2). In contrast, treatment with perillaldehyde at 0.5×MIC induced significant morphological alterations, including hyphal wrinkling, shrinkage, and localized disintegration. These structural disruptions indicate substantial impairment of normal mycelial development. Notably, similar morphological aberrations have been documented in *A. carbonarius* following exposure to *Eremanthus erythropappus* essential oil and eugenol [18,20], suggesting a common mechanism of action among these antifungal agents [18].

### 2.3. Effects of Perillaldehyde on Cell Integrity of A. carbonarius

**Figure 3:**
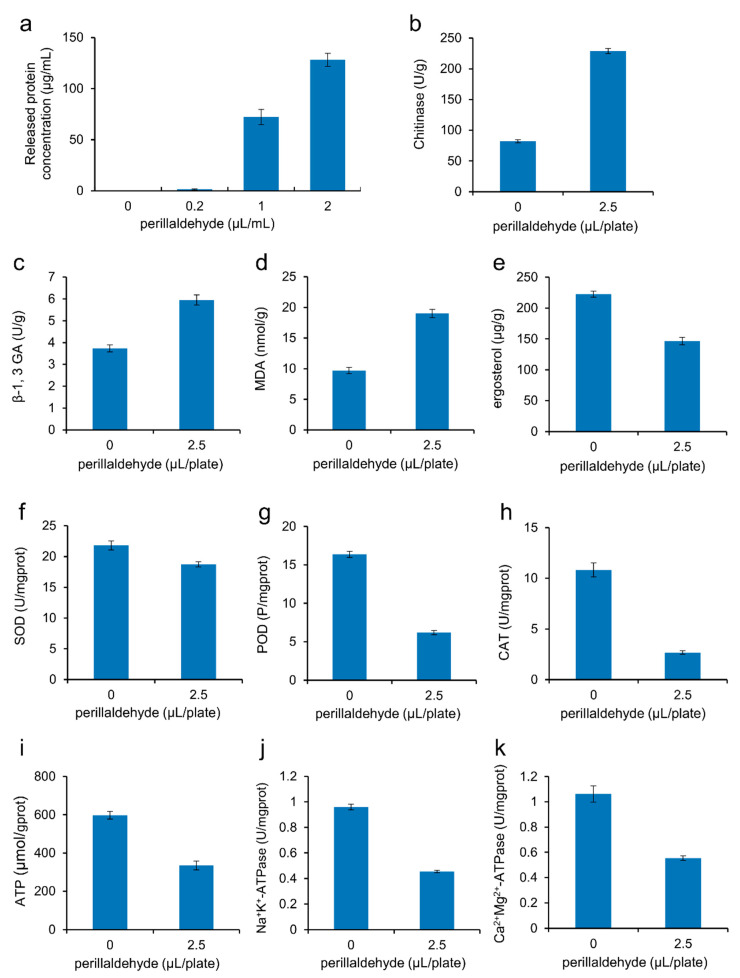
Effects of perillaldehyde on the intracellular protein leakage from spores of *A. carbonarius* (**a**), activities of chitinase (**b**), β-1,3-glucanase (**c**), SOD (**f**), POD (**g**), CAT (**h**), Na^+^K^+^-ATPase (**j**), and Mg^2+^Ca^2+^-ATPase (**k**) in the cells of *A. carbonarius*, and contents of malondialdehyde (**d**), ergosterol (**e**) and ATP (**i**) in the cells of *A. carbonarius*. MDA: malondialdehyde; SOD: superoxide dismutase; POD: peroxidase; CAT: catalase; ATP: adenosine triphosphate; ATPase: adenosine triphosphatase. Data are expressed as mean ± standard error (*n* = 3).

This study evaluated fungal membrane integrity by quantifying intracellular protein leakage from *A. carbonarius* spores following perillaldehyde treatment. While control samples showed negligible extracellular protein levels, exposure to 0.2, 1, and 2 µL/mL perillaldehyde induced dose-dependent protein release from 1.5 to 128.2 μg/mL (Figure 3a). These findings demonstrate a strong correlation between protein leakage and concentration-dependent membrane disruption (R^2^ = 0.99, *p* < 0.01). The substantial loss of intracellular proteins at higher concentrations (≥1 µL/mL) suggests severe membrane compromise, directly implicating perillaldehyde-induced cellular damage as a primary mechanism of fungicidal activity [19].

The fungal cell wall is crucial for maintaining the inherent morphology and structural integrity of fungal cells. It is a complex structure made of polysaccharides, including chitin and glucan [21,22]. Chitinase can hydrolyze chitin glycosidic bonds, while β-1,3-glucanase can hydrolyze β-1,3-glucosidic linkages. Therefore, both chitinase and β-1,3-glucanase can cause the degradation of the fungal cell wall, hyphal autolysis, and spore separation [23,24]. To better illustrate the mechanisms for the cell rupture, the activities of chitinase and β-1,3-glucanase were measured. Perillaldehyde treatment at 0.5×MIC significantly enhanced cell wall-degrading enzyme activities in *A. carbonarius*, with chitinase activity increasing to 2.8-fold (from 82.5 U/g to 232.3 U/g; Figure 3b) and β-1,3-glucanase activity increasing to 1.6-fold (from 3.7 U/g to 6.0 U/g; Figure 3c). Thus, perillaldehyde can increase the activities of chitinase and β-1,3-glucanase, thereby reducing the contents of chitin and glucan in *A. carbonarius* cell walls. The findings align with those of previous research, according to which natural antifungal agents can cause obvious degradation of the fungal cell wall [25,26].

Malondialdehyde (MDA) is the main biomarker and product of lipid peroxidation. Elevated levels of MDA suggest a damaged cell membrane [27]. When *A. carbonarius* is treated with 0.5×MIC of perillaldehyde, its MDA content increases from 9.7 nmol/g to 19.0 nmol/g, an increase of 1.96 times (Figure 3d). Therefore, it is indicated that the degree of lipid peroxidation in *A. carbonarius* cells increased under the stress of perillaldehyde. This result is similar to that of previous reports, which suggest that natural antifungal agents can cause lipid peroxidation in fungal cells, altering the functionality and integrity of the cell membrane, and producing excessive MDA [27,28].

Ergosterol is of vital importance in the constitution of fungal cell membranes and serves as an indicator of fungal biomass [22,29]. When *A. carbonarius* is treated with 0.5×MIC of perillaldehyde, its ergosterol content decreases from 222.5 μg/g to 146.6 μg/g (Figure 3e). This result is consistent with the inhibition of ergosterol biosynthesis in *A. carbonarius* after *Eremanthus erythropappus* EO or eugenol treatment [18,20], indicating that lipophilic perillaldehyde can disintegrate the cell membrane by inhibiting the biosynthesis of ergosterol.

### 2.4. Effects of Perillaldehyde on Mycelial Antioxidant Enzymatic Activities of A. carbonarius

The maintenance of cellular oxidative homeostasis relies on the dynamic equilibrium between the generation and removal of reactive oxygen species (ROS). All organisms, under normal conditions, contain a complete set of antioxidant systems to remove free radicals from their bodies. Consequently, the free radicals and the oxidation–reduction balance of cells are maintained at an appropriate level. However, when cells encounter stress, this balance is disturbed, leading to oxidative damage and cell death. This is one of the potential mechanisms by which plant essential oils exert their antifungal effects [27]. Superoxide dismutase (SOD), catalase (CAT), and peroxidase (POD) are essential for the fungal cellular antioxidant defenses [30]. SOD is a major antioxidant enzyme capable of clearing superoxide anions in mitochondria [31]. CAT is a hallmark enzyme of the peroxisome involved in peroxide metabolism. The main role of CAT is to catalyze the decomposition of H_2_O_2_ into O_2_ and H_2_O [32]. POD utilizes H_2_O_2_ as an electron acceptor to catalyze substrate oxidation. Additionally, it detoxifies H_2_O_2_ along with phenols, amines, and aldehydes [33]. This dual functionality underscores its critical role in fungal antioxidant defense. We found that the treatment of *A. carbonarius* with 0.5×MIC of perillaldehyde decreased SOD (Figure 3f), POD (Figure 3g), and CAT (Figure 3h) activities by 14.2%, 62.2%, and 75.3%, respectively. These results indicated that perillaldehyde impaired the antioxidant system of *A. carbonarius*, weakened the cells’ ability to clear ROS, and caused strong oxidative damage to *A. carbonarius*. A previous investigation has demonstrated that the application of Perilla oil decreases the levels of SOD, CAT, and POD in *A. flavus* [34]. The aldehyde moiety on the molecular structure of perillaldehyde can react with some protein groups, such as -SH (sulfhydryl group) and -NH_2_ (amino group), thereby inactivating some proteins. This behavior of perillaldehyde explains the damage caused to the fungal antioxidant system.

### 2.5. Effects of Perillaldehyde on the Energy Metabolism of A. carbonarius

Energy metabolism is crucial for fungal growth. While perillaldehyde has been shown to induce cell death in *A. flavus* [35], its effects on the energy metabolism of *A. carbonarius* remain unexplored. Adenosine triphosphate (ATP) serves as a key energy carrier, and fluctuations in ATP levels can significantly impact cellular function. ATPase catalyzes the hydrolysis of ATP into adenosine diphosphate (ADP) and phosphate ions, a process critical for material transport, information transmission, and energy conversion [27]. For example, the Na^+^K^+^-ATPase located in the cell membrane helps exchange intracellular sodium with extracellular potassium against the concentration gradient, and Mg^2+^Ca^2+^-ATPase can transfer calcium and magnesium ions [36]. These indicators are important for studying the impact of antifungal agents on the energy metabolism of fungi. To evaluate the impact of perillaldehyde treatment on the energy metabolism of *A. carbonarius*, the ATP levels and the activities of Na^+^K^+^-ATPase and Mg^2+^Ca^2+^-ATPase in different treatment groups were measured. In comparison to the control group, there was a 44.0% decrease in ATP contents in the treatment groups (Figure 3i), indicating that the post-perillaldehyde treatment mitochondrial capacity of *A. carbonarius* may be insufficient. Similar to changes in the ATP content, the post-perillaldehyde enzymatic activities of Na^+^K^+^-ATPase and Mg^2+^Ca^2+^-ATPase decreased by 52.7% (Figure 3j) and 47.9% (Figure 3k), respectively, indicating that perillaldehyde can effectively suppress the energy metabolism of *A. carbonarius*. Ju et al. also found that Perilla oil could significantly decrease the ATP levels in *A. flavus* and suppress the activities of ATPase [27].

### 2.6. Comprehensive Transcriptomic Profile Analysis

Transcriptome analysis was carried out to investigate the possible antifungal and antimycotoxigenic molecular mechanisms of perillaldehyde against *A. carbonarius* at the mRNA level. To our knowledge, this study presents the first transcriptional reaction profile of *A. carbonarius* exposed to perillaldehyde treatment. RNA-Seq analysis yielded an average of 35.4 million clean reads from control mycelia and 35.7 million from mycelia treated with 0.5×MIC of perillaldehyde on PDA medium. A total of 1103 genes were identified as differentially expressed genes (DEGs), with 391 genes showing significant up-regulation and 712 genes showing significant down-regulation.

**Figure 4:**
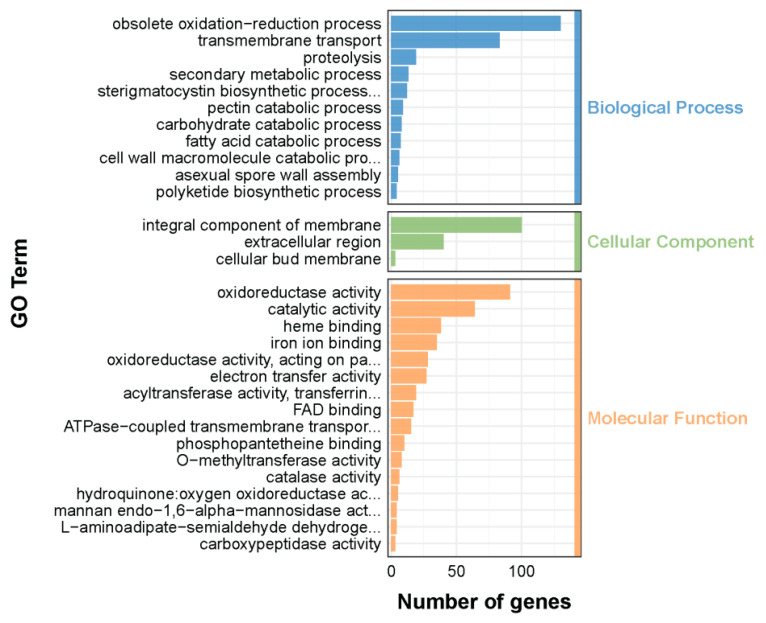
Functional categorization of differentially expressed genes (DEGs) via Gene Ontology (GO). The top 30 enriched results are listed in descending sequence according to the number of genes in each of the three domains.

**Figure 5:**
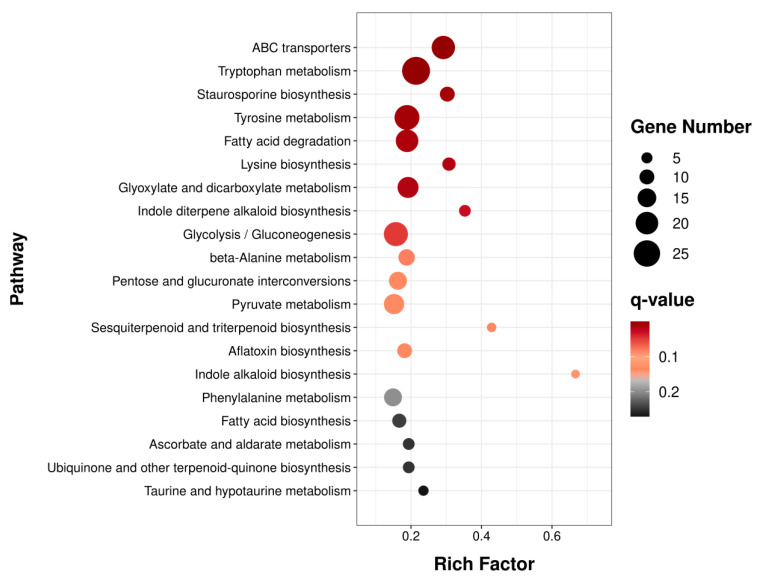
Kyoto Encyclopedia of Genes and Genomes (KEGG) pathways of DEGs. The top 20 most significantly enriched results are presented in ascending order based on the *q*-value.

The functions, metabolic pathways, as well as the interrelationships of these DEGs were further determined by Gene Ontology (GO) and Kyoto Encyclopedia of Genes and Genomes (KEGG) enrichment analyses. Results of the GO enrichment analysis of the DEGs revealed significant functional annotations among the top 30 GO terms. In the biological process category, oxidation-reduction process, transmembrane transport, and proteolysis were prominently enriched. For cellular components, the most enriched terms included integral membrane components, extracellular regions, and cellular bud membranes. Within molecular functions, oxidoreductase activity, catalytic activity, and heme binding showed significant enrichment (Figure 4). Additionally, the DEGs were grouped into metabolic pathways through KEGG enrichment analysis. Among these pathways, ABC transporters, amino acid metabolism (tryptophan metabolism pathway, tyrosine metabolism pathway, and lysine biosynthesis pathway, etc.), and fatty acid degradation turned out to be the pathways with the highest enrichment level (Figure 5). Moderate enrichment was observed in carbohydrate metabolic pathways, including glyoxylate and dicarboxylate metabolism, glycolysis/gluconeogenesis and pyruvate metabolism. Similarly, pathways involved in secondary metabolite biosynthesis, particularly aflatoxin biosynthesis, also showed moderate enrichment.

The GO enrichment analysis revealed that 164 DEGs were enriched for the term oxidation-reduction process (GO:0055114) or oxidoreductase activity (GO:0016491). Therein, 95 genes were down-regulated and 69 genes were up-regulated. For instance, most of the catalase genes were significantly down-regulated in response to perillaldehyde treatment, including ASPCADRAFT_153376, ASPCADRAFT_174418, ASPCADRAFT_210875, ASPCADRAFT_518556 and ASPCADRAFT_9402. Similar conclusions were obtained for *A. carbonarius* treated by eugenol [18]. Normally, oxidation-reduction reactions can promote fungal metabolism and biological adaptation to the environment. Changes in the oxidation-reduction process and related enzyme activities indicated that perillaldehyde could interfere with the metabolic process of *A. carbonarius* by altering its oxidation-reduction reactions. This is consistent with the results that MDA content increased, total antioxidant capacity decreased, and antioxidant enzymatic activities decreased. These results suggested that the intracellular environment of *A. carbonarius* was in an oxidation-reduction imbalance state under the oxidative stress caused by perillaldehyde.

The fungal cell wall is a potential molecular target for antifungal agents [37]. In this study, significant down-regulation of the chitin synthase (CHS1) gene (ASPCADRAFT_398403) would lead to decreased chitin content. The chitinase genes (ASPCADRAFT_209247, ASPCADRAFT_135036, ASPCADRAFT_172228, and ASPCADRAFT_206965) were significantly up-regulated, indicating that chitin degradation was further enhanced and chitin loss was aggravated. Previous research has shown that the down-regulation of a gene related to glucan synthesis (ASPCADRAFT_126516) under external stress may be associated with the decrease in glucan content in fungal cells [22], a finding that was confirmed in the present study. These results further indicated that perillaldehyde damages the integrity of the cell wall at the molecular level.

When microorganisms are subjected to adverse stimuli, they activate their self-protection system and alter the fatty acid constitution of the cell membrane to maintain its fluidity [19]. Ergosterol, fatty acids, as well as unsaturated fatty acids contribute to the fluidity and integrity of cell membranes [22]. Sterol 14α-demethylase (CYP51) is a key enzyme in the sterol synthesis process, particularly in ergosterol biosynthesis, and the lack of this enzyme can result in the loss of membrane structure and function. Consequently, inhibition of CYP51 has emerged as a critical target of antifungal agents [38]. In this study, the CYP51 gene (ASPCADRAFT_166660) was significantly down-regulated. *ERG26* is the encoding gene for sterol-4α-carboxylate 3-dehydrogenase and is essential for ergosterol synthesis and fungal growth [39]. The ERG26 gene (ASPCADRAFT_133622) was down-regulated, further confirming that perillaldehyde can cause a decrease in ergosterol content in *A. carbonarius*.

The tricarboxylic acid (TCA) cycle is an important energy metabolism pathway in eukaryotic mitochondria. Citrate synthase catalyzes the synthesis of citric acid by condensing acetyl-CoA and oxaloacetic acid and is the rate-limiting enzyme in the TCA cycle. In this research, the encoding gene of citrate synthase (ASPCADRAFT_212258) underwent significant down-regulation, suggesting that perillaldehyde could lead to the stagnation or interruption of the TCA cycle, which may affect the energy metabolism of *A. carbonarius* cells. In addition, the ATP biosynthesis-related gene (ASPCADRAFT_508118) was significantly down-regulated, which may lead to a reduction in ATP content. This suggests that perillaldehyde may damage the mitochondria, resulting in insufficient energy supply to the cells of *A. carbonarius*.

Amino acid metabolism plays a vital role in the growth and development of fungi. In this study, many down-regulated DEGs were enriched in amino acid metabolism pathways including the tryptophan metabolism pathway, tyrosine metabolism pathway, and lysine biosynthesis pathway. These findings suggested that perillaldehyde might inhibit the growth and development of *A. carbonarius* by affecting its amino acid metabolism. In addition, the transport of amino acids is essential for the regulation of amino acid metabolism. Four DEGs (ASPCADRAFT_209197, ASPCADRAFT_208820, ASPCADRAFT_202690, and ASPCADRAFT_10893), which were enriched for the term amino acid transmembrane transport (GO:0003333) and amino acid transporter activity (GO:0015171), were significantly down-regulated in this study, indicating that perillaldehyde may lead to impaired function of amino acid transporter in *A. carbonarius*, thus affecting the synthesis of related proteins. This impairment also disrupts intracellular amino acid uptake and transport, leading to dysfunction of metabolic and physiological processes dependent on amino acids.

The secondary metabolic gene clusters of fungi often regulate the expression of related genes through different levels of transcriptional regulatory elements, and there are many genes and proteins directly or indirectly involved in the synthesis of mycotoxins, including global regulatory genes, signal transduction genes, and transporters [40]. The transporters mainly consist of ATP-binding cassette (ABC), and the major facilitator superfamily (MFS) transporters. As efflux pumps, transporters avoid toxic effects by squeezing out toxic metabolites from microorganisms [41]. In this study, after being treated with perillaldehyde, the DEGs of *A. carbonarius* were significantly enriched in the pathway of ABC transporters, indicating that the active transport process of substances was changed, and various life activities such as heavy metal detoxification, lipid metabolism, signal transduction, and multidrug resistance were affected [42]. The ABC transporter gene (ASPCADRAFT_211441) of *A. carbonarius* is homologous to *atrF*, the efflux pump gene of *A. fumigatus*, and the drug resistance transporter gene (ASPCADRAFT_133982) is homologous to the effector pump gene *Cdr4* in *Neurospora crassa* [43]. The two genes were significantly up-regulated in this study, indicating that *A. carbonarius* can reduce the accumulation of perillaldehyde in cells by increasing drug efflux. ABC transporters can also affect the transport of mycotoxins in fungi. A previous study has shown that the multidrug-resistant protein Pdr5p, belonging to the ABC transporter superfamily, in *Saccharomyces cerevisiae* can affect the efflux of deoxynivalenol (DON) and 15-DON [44]. Whether the relevant ABC transporter in this study is related to OTA transport efflux requires further investigation.

In addition, studies have shown that MFS transporters are involved in the transportation of mycotoxins. For example, the *otatraPN* gene, found in *Penicillium nordicum*, plays a role in the secretion and transport of OTA [45]. The expression of the MFS transporter Flu1 is notably high in *A. carbonarius* strains with high levels of OTA [46,47], indicating its potential involvement in the secretion and transport of OTA. Its homologous gene in *A. carbonarius* (ASPCADRAFT_135554, *AcFlu1*) was significantly down-regulated in our study. The *aflT* gene, located in the cluster of genes responsible for aflatoxin biosynthesis, encodes an MFS family fungal transporter associated with the efflux and transport of aflatoxins [48]. Its homologous gene in *A. carbonarius* (ASPCADRAFT_173225, *AcaflT*) is also significantly down-regulated. These findings indicated that perillaldehyde may hinder the secretion and transport of OTA in *A. carbonarius* by reducing the expression levels of genes related to transport proteins. Beyond indirectly suppressing OTA production by inhibiting the growth of *A. carbonarius*, perillaldehyde directly interferes with OTA biosynthesis and transport at the genetic level. This dual mechanism explains its potent inhibitory effect on OTA production in *A. carbonarius.*

**Figure 6:**
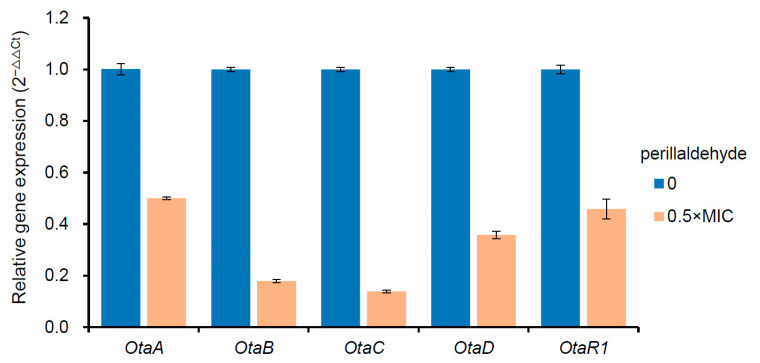
Effects of perillaldehyde on the expression of OTA biosynthetic cluster genes including *OtaA*, *OtaB*, *OtaC*, *OtaD and OtaR1*, as analyzed by reverse transcription-quantitative polymerase chain reaction (RT-qPCR). Data are expressed as mean ± standard error (*n* = 3).

Previous studies have established that OTA biosynthesis begins with polyketide synthases (OtaA), which combine acetyl-CoA and malonyl-CoA to synthesize 7-methylsclerotiorin. This intermediate is subsequently oxidized by OtaC to yield Otβ, which then undergoes amide bond formation with L-β-phenylalanine via OtaB, producing ochratoxin B (OTB). The final step involves OtaD-mediated chlorination of OTB to generate OTA [16]. OtaR1 is a pathway-specific regulator that controls OTA production by regulating the above four biosynthetic genes, and OtaY plays an essential role in polyketone cyclization in the initial stage of OTA biosynthesis [16,17]. A further analysis in this study showed that the expressions of the genes of *OtaA* (ASPCADRAFT_173482), *OtaB* (ASPCADRAFT_132610), *P450* (ASPCADRAFT_517149, *OtaC*), *OtaD* (ASPCADRAFT_209543), *OtaY* (ASPCADRAFT_209537), and *bZIP* (ASPCADRAFT_7821, *OtaR1*) were significantly down-regulated under the stress of perillaldehyde (Table 1). These results suggest that perillaldehyde can directly inhibit the production of OTA by impairing the transcriptional activity of all the known conserved genes involved in OTA biosynthesis. Previous research has shown that natural antifungal agents can regulate the expression of mycotoxin biosynthetic genes in fungi. For instance, the transcription levels of OTA*pks* and OTA*nrps* genes in *A. ochraceus* can be inhibited by cinnamaldehyde [16]. In order to validate the accuracy of RNA-Seq data, the expression levels of *OtaA*, *OtaB*, *OtaC*, *OtaD*, *and OtaR1* genes in the OTA biosynthetic gene cluster were analyzed through reverse transcription-quantitative polymerase chain reaction (RT-qPCR). The results demonstrated that the expression levels of the aforementioned five genes were significantly lowered compared to the control group (Figure 6), implying that the transcriptome analysis is a stable and reliable methodology.

## 3. Conclusions

**Figure 7:**
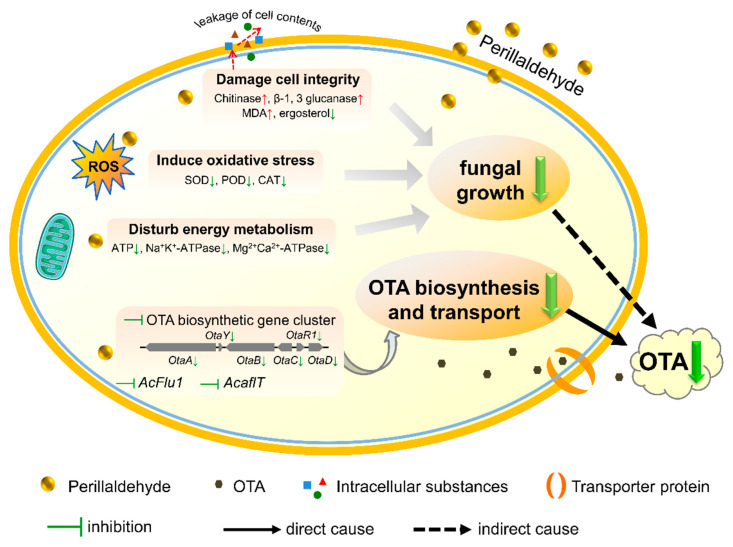
Proposed mechanisms of perillaldehyde-mediated inhibition of OTA biosynthesis.

This study demonstrated that perillaldehyde exhibits dose-dependent inhibition of *A. carbonarius* growth and OTA production both *in vitro* and *in vivo*. A comprehensive analysis (Figure 7) of fungal growth parameters, metabolic indicators, and transcriptome data revealed multiple antifungal mechanisms: (1) disruption of cellular integrity, (2) induction of oxidative stress, and (3) interference with energy metabolism, which collectively contribute to indirect OTA suppression. Furthermore, perillaldehyde directly inhibits OTA biosynthesis and transport through the downregulation of key biosynthetic genes. These results illustrate how perillaldehyde modulates the intrinsic antifungal pathways of *A. carbonarius* and interferes with OTA production. However, there are still some limitations to this study, for example, the underlying molecular mechanisms, including upstream signaling molecules and key protein targets in *A. carbonarius*, require further investigation. Additionally, the experiments were conducted under laboratory conditions, so their efficacy in actual grape storage environments remains unvalidated, and the optimal dose in grapes and long-term residual risks still require evaluation. Given its dual inhibitory effects on fungal proliferation and mycotoxin production, perillaldehyde represents a promising natural alternative for controlling *A. carbonarius* contamination and OTA accumulation in grapes and grape-derived products, offering both antifungal and antimycotoxigenic benefits.

## 4. Materials and Methods

### 4.1. Strain and Culture Condition

A strain of *A. carbonarius* (CICC 41254) was acquired from the China Center of Industrial Culture Collection (CICC) and incubated on PDA medium at 25 °C for 6 days. Spore suspensions were then prepared according to a previously published method [18]. The concentration of spores was adjusted to 1 × 10^6^ spores per milliliter using an aqueous solution of Tween-80 (0.1%, *v*/*v*) for the subsequent experiments.

### 4.2. Analysis of the Antifungal and Antimycotoxigenic Effects of Perillaldehyde on A. carbonarius, In Vitro and In Vivo

*In vitro*, the spore suspensions of *A. carbonarius* were inoculated on one side of a two-cell Petri plate containing the PDA medium. Different doses (0, 0.625, 1.25, 2.5, and 5 µL/plate) of perillaldehyde were added to the other side. Another Petri plate with no perillaldehyde was employed as the experimental control. The fungal colony was incubated at 25 °C for 6 days, after which its diameter was measured. The mycelia were then collected and ground to a fine powder with liquid nitrogen for subsequent experiments, including the determination of enzyme activities, metabolite analysis, and RNA extraction. Each test was performed independently at least three times. *In vivo*, the grapes (*V. vinifera* L. × *V. labrusca* L. ‘Kyoho’) were obtained from a picking garden located in Daxing District, Beijing. Grape berries of uniform size, consistent maturity, and without any mechanical damage were selected. They were first sterilized and dried and then randomly divided into five groups, with 30 grape berries in each group. A 2-mm-diameter and 3-mm-deep hole was made in each berry. Each of these holes was filled with 5 μL of a 1 × 10^6^ spores/mL *A. carbonarius* spore suspension. The processed berries were separately placed in five sealable plastic boxes, which were then supplied with different doses of perillaldehyde (0, 5, 10, 20, and 40 μL/L) individually. Grapes without perillaldehyde were employed as the control group, while grapes fumigated with different concentrations of perillaldehyde were set as the treatment group. The treatment group was placed at 25 °C for 3 days and was followed by measurement of the disease spot diameter using the cross method. The tissue around the lesion was removed with the help of an aseptic scalpel and frozen in liquid nitrogen to determine the OTA levels. Each experiment consisted of three parallel treatments. The MIC was determined as the lowest concentration of perillaldehyde that inhibited observable mycelial growth. Calculation of growth inhibition was performed using the following equation: Inhibition ratio (%) = (*D*_c_ − *D*_t_)/*D*_c_ × 100. *D*_c_ represents the colony diameter of the control sample and *D*_t_ is the colony diameter of the sample treated with perillaldehyde. All tests were performed in triplicate. OTA was extracted from the PDA medium and determined in triplicate using a method that has been described previously [18].

### 4.3. Scanning Electron Microscopy (SEM) Analysis

The morphology of the *A. carbonarius* mycelium treated with perillaldehyde at concentrations of 0 and 2.5 µL/plate, on PDA medium, was observed by SEM according to the previous report [19]. Briefly, *A. carbonarius* was cultivated on PDA medium for 48 h, and then the mycelia and spores were harvested and rinsed twice with 0.1 M phosphate-buffered saline (PBS). After fixation and gradient dehydration, the collected samples were placed in tert-butanol and then dried through freeze-drying. Then, they were mounted on a stub and coated with gold. Finally, the micromorphology of the samples was examined using SEM (S-3400N, Hitachi, Tokyo, Japan).

### 4.4. Intracellular Protein Release

Leakage of proteins from the spores of *A. carbonarius*, after being cultured for 7 days with perillaldehyde, was detected using a previously described method [18], with some adjustments. In brief, the spores were collected and rinsed with sterile, double-distilled water through centrifugation. They were then suspended in a sterile phosphate buffered saline (0.01 M, pH 7.2–7.4) containing different concentrations of perillaldehyde (0, 0.2, 1, and 2 µL/mL) at a concentration of 1 × 10^6^ spores/mL and allowed to incubate at 25 °C for 24 h. The total amount of leaked protein content was quantified using a bicinchoninic acid protein assay kit from Beijing Solarbio Science & Technology Co., Ltd., Beijing, China.

### 4.5. Analysis of Metabolism-Related Enzymatic Activities and Substances

The mycelia of *A. carbonarius*, which were treated with perillaldehyde at concentrations of 0 and 2.5 µL/plate and incubated at 25 °C for 6 days on PDA medium as described in Section 2.2, were harvested and thoroughly ground with liquid nitrogen to determine the metabolism-related enzymatic activities and substances. The total amounts of ATP and MDA, and the enzymatic activities of POD, SOD, CAT, β-1,3-glucanase, chitinase, Na^+^K^+^-ATPase, and Mg^2+^Ca^2+^-ATPase were determined according to the instructions of the respective detection kits purchased from Beijing Solarbio Science & Technology Co., Ltd. (Beijing, China), accompanied by an EnVision Multilabel plate reader (PerkinElmer, Boston, MA, USA). The ergosterol level was measured using the previously established method [19].

### 4.6. Transcriptome Analysis

For transcriptomic analysis, the mycelia of *A. carbonarius* treated with perillaldehyde at 0 and 2.5 µL/plate were harvested from the PDA plates described in Section 2.2 and subsequently finely ground using liquid nitrogen. Total RNA was extracted using TRIzol reagent (Thermo Fisher, Waltham, MA, USA) following the manufacturer’s protocol. The total RNA quantity, purity and integrity were evaluated by a Bioanalyzer 2100 and RNA 6000 Nano LabChip Kit (Agilent, Santa Clara, CA, USA), and high-quality RNA samples with RIN number >7.0 were used to construct a sequencing library. mRNA was purified from total RNA using Dynabeads Oligo (dT) (Thermo Fisher, Waltham, MA, USA) with two rounds of purification. Two cDNA strands were generated after cleavage using NEBNext^®^ Magnesium RNA Fragmentation Module (New England Biolabs, Ipswich, MA, USA). The average insert size for the final cDNA library was 300 ± 50 bp. Finally, 2 × 150 bp paired-end sequencing (PE150) was performed on an Illumina Novaseq™ 6000 platform (LC-Bio Technology CO., Ltd., Hangzhou, China) following the vendor’s recommended protocol. The clean reads were obtained by filtering the raw reads and were subsequently aligned to the reference genome of *A. carbonarius*, using HISAT2 (https://daehwankimlab.github.io/hisat2/, version: 2.2.1) package. The mapped reads of each sample were assembled by StringTie (http://ccb.jhu.edu/software/stringtie/, version: 2.1.6) with default parameters. Then all transcriptomes from all samples were merged to reconstruct a comprehensive transcriptome using gffcompare (http://ccb.jhu.edu/software/stringtie/gffcompare.shtml, version: 0.9.8). StringTie and ballgown (http://www.bioconductor.org/packages/release/bioc/html/ballgown.html) were used to estimate the expression levels of all transcripts and analyze expression abundance of mRNAs by calculating FPKM (fragment per kilobase of transcript per million mapped reads). The genes with a |log2 Fold Change| ≥ 1 and a *q*-value < 0.05 were identified as DEGs. GO enrichment and KEGG pathway enrichment analysis were employed to further characterize the DEGs.

### 4.7. Gene Expression Analysis by Reverse Transcription-Quantitative Polymerase Chain Reaction (RT-qPCR)

Total RNA of *A. carbonarius* was extracted from the perillaldehyde-treated (0 and 2.5 µL/plate) mycelia obtained from PDA medium using TRIzol^TM^ reagent. The RNA was then analyzed by RT-qPCR using primer sequences designed as described previously [18]. Reverse transcription was performed using the TransScript II First-strand cDNA Synthesis SuperMix kit (TransGen Biotech, Beijing, China) to synthesize the cDNA. RT-qPCR was carried out on the LightCycler^®^96 system (Roche Molecular Systems, Inc., Pleasanton, CA, USA), and the 2^−ΔΔCt^ method was used to determine the relative transcription levels of the genes.

### 4.8. Data Availability

The raw sequence data reported in this paper have been deposited in the Genome Sequence Archive (GSA) [49] at the National Genomics Data Center (NGDC) [50], China National Center for Bioinformation (CNCB)/Beijing Institute of Genomics, Chinese Academy of Sciences (GSA: CRA022489), which is publicly accessible at https://ngdc.cncb.ac.cn/gsa (accessed on 22 January 2025).

### 4.9. Statistical Analysis

The data analysis was conducted by means of the IBM SPSS Statistics 23.0 software (IBM Inc., Armonk, NY, USA). A significance level of *p* < 0.05 was adopted.

## Figures and Tables

**Table 1:** A comparison of representative differentially expressed genes (DEGs) involved in OTA biosynthesis and transport in *Aspergillus carbonarius* between perillaldehyde treatment and control.

Gene ID	log_2_(PA/CK)	*q*-Value	Description
ASPCADRAFT_173482	−1.832	0.000	OTA*pks*, Ochratoxin A biosynthesis cluster protein A (OtaA)
ASPCADRAFT_209537	−4.863	0.000	OTA*cyc*, probable cyclase, Ochratoxin A biosynthesis cluster protein Y (OtaY)
ASPCADRAFT_132610	−3.353	0.000	OTA*nrps*, Ochratoxin A biosynthesis cluster protein B (OtaB)
ASPCADRAFT_517149	−4.052	0.000	*P450*, Cytochrome P450 monooxygenase, Ochratoxin A biosynthesis cluster protein C (OtaC)
ASPCADRAFT_7821	−2.387	0.001	*bZIP*, Transcription factor, Ochratoxin A biosynthesis cluster protein R1 (OtaR1)
ASPCADRAFT_209543	−3.007	0.000	OTA*hal*, Flavin-dependent halogenase, Ochratoxin A biosynthesis cluster protein D (OtaD)
ASPCADRAFT_135554	−5.220	0.000	Homology with the *Flu1* gene, which is involved in OTA secretion and transport
ASPCADRAFT_173225	−1.370	0.007	Homology with the *aflT* gene, which is associated with the exogenesis and transport of aflatoxins

## Data Availability

The raw sequence data presented in this study have been deposited in the Genome Sequence Archive in National Genomics Data Center, China National Center for Bioinformation/Beijing Institute of Genomics, Chinese Academy of Sciences that is publicly accessible at https://ngdc.cncb.ac.cn/gsa, reference number [CRA022489]. [Genome Sequence Archive] [https://ngdc.cncb.ac.cn/gsa] [CRA022489].
